# Virtual Care in Rhinology

**DOI:** 10.1186/s40463-021-00505-1

**Published:** 2021-04-13

**Authors:** Kristine A. Smith, Andrew Thamboo, Yvonne Chan, Christopher J. Chin, Megan Werger, Brian Rotenberg

**Affiliations:** 1 Department of Otolaryngology - Head and Neck Surgery, University of Manitoba, GB421B – 820 Sherbrook Street, Winnipeg, Manitoba Canada; 2 Division of Otolaryngology – Head & Neck Surgery, Department of Surgery, University of British Columbia, Vancouver, Canada; 3 Department of Otolaryngology – Head & Neck Surgery, University of Toronto, Toronto, Canada; 4 Divsion of Otolaryngology-Head and Neck Surgery, Dalhousie University, Saint John, NB Canada; 5 McMaster University, Toronto, Canada; 6 Department of Otolaryngology – Head and Neck Surgery, Schulich School of Medicine and Dentistry, Western University, London, Canada

**Keywords:** Virtual care, Rhinology, COVID-19, Coronavirus

## Abstract

**Background:**

The SARS-CoV-2 (COVID) pandemic has resulted in an increase in virtual care. While some specialties are well suited to virtual care, Otolaryngology – Head and Neck Surgery could be limited due to reliance on physical examination and nasal endoscopy, including Rhinology. It is likely virtual care will remain integrated for the foreseeable future and it is important to determine the strengths and weaknesses of this treatment modality for rhinology.

**Methods:**

A survey on virtual care in rhinology was distributed to 61 Canadian rhinologists. The primary objective was to determine how virtual care compared to in-person care in each area of a typical appointment. Other areas focused on platforms used to deliver virtual care and which patients could be appropriately assessed by virtual visits.

**Results:**

43 participants responded (response rate 70.5%). The majority of participants use the telephone as their primary platform. History taking and reviewing results (lab work, imaging) were reported to be equivalent in virtual care. Non-urgent follow up and new patients were thought to be the most appropriate for virtual care. The inability to perform exams and nasal endoscopy were reported to be significant limitations.

**Conclusion:**

It is important to understand the strengths and limitations of virtual care. These results identify the perceived strengths and weaknesses of virtual care in rhinology, and will help rhinologists understand the role of virtual care in their practices.

**Graphical abstract:**

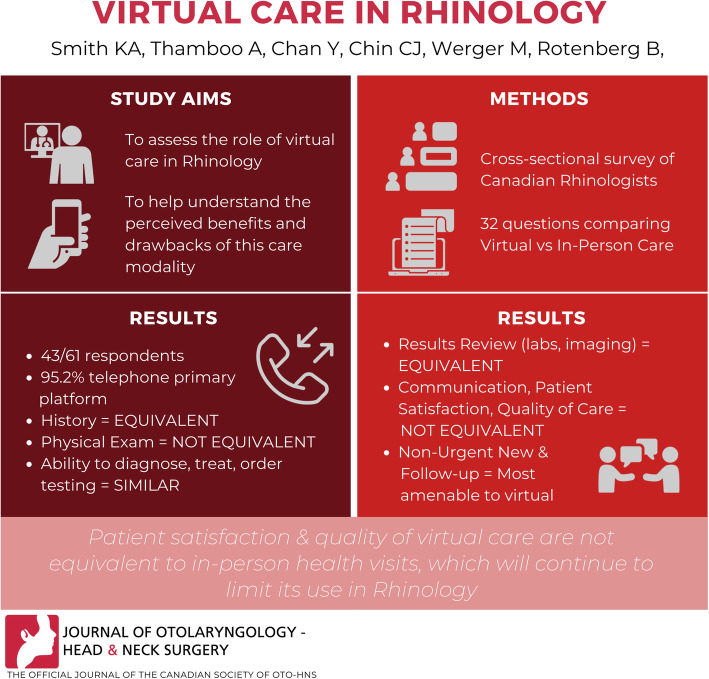

## Introduction

With the emergence of the SARS-CoV-2 virus in December 2019 and the associated pandemic declared in March 2020, there was a rapid transition from in-person appointments to virtual care for many medical practices [1]. Locoregional lockdowns and restrictions on in-person appointments necessitated another form of care to ensure patients were affected as minimally as possible. Virtual visits provided an alternative to in-person care. For many, this was seen as a temporary measure that would cease when the SARS-CoV-2 outbreak subsided. Unfortunately, it appears the current coronavirus is not going to dissipate like its predecessors, SARS-CoV-1 and MERS. Subsequently physical distancing and restrictions on in-person visits will likely be present for the foreseeable future.

The need for physical distancing in hospitals and private clinics has resulted in a significant reduction in the availability of in-person appointments. Virtual visits can help balance the need to reduce in-person practice volumes and meet the needs of patients awaiting new consultations and follow up care. While telemedicine has been available for years, its use is generally limited to patients who live in remote or difficult to access areas. Now, virtual care is nearly ubiquitous in North America. There are many areas in medicine which are well suited to virtual care. However, Otolaryngology – Head and Neck Surgery (OtoHNS) is a specialty that often relies on physical examination to aid in diagnosis. Some components of the physical exam may be possible through various virtual platforms, but there is currently no alternative for endoscopy, an important component of an otolaryngologic assessment. As a result, virtual visits may limit an Otolaryngologist’s ability to accurately diagnose and manage disease.

Additional outbreaks of SARS-CoV-2 virus are already occurring in some areas of the world. As these viral outbreaks ebb and flow, transitions between a state of lockdown and cautious reopening are expected. Virtual care will likely play a significant role in physicians’ ability to provide continuity of care during times of greater restrictions, and will likely be present in some form as long as physical distancing is needed. Otolaryngology is a diverse subspecialty, which treats a highly varied complement of disease. Within Rhinology, a subspecialty of Otolaryngology, there are some circumstances where virtual care may be comparable to traditional in-person care and others where it may be less effective. The goal of this study is to assess the opinions of the role of virtual care in Rhinology, to help understand the perceived benefits and drawbacks of this care modality.

## Methods

### Study design and subjects

This study was a cross-sectional survey of Canadian rhinologists. Participants were identified from an email list from the Canadian Rhinology Working Group. Inclusion criteria were Canadian Otolaryngologists with fellowship training in rhinology or an emphasis in their practice on rhinology.

A 32-question survey was developed by the authors, which primarily focused on determining how virtual care compared to in-person care in each area of a typical appointment (history, physical exam, diagnosis, treatment, etc). Other areas of the survey focused on platforms used to deliver virtual care and which presenting complaints were thought to be equally assessed by a virtual visit compared to in-person assessments. The survey is available for review in supplemental material 1. Following development of the survey, it was inputted into SurveyMonkey (San Mateo, California, 1999–2020). In June 2020, an initial email invitation to the survey was distributed. Two subsequent reminder emails were sent approximately 1 week apart. Participation was voluntary and no identifying data was collected, including IP addresses. The survey was closed after 4 weeks of collecting responses. Survey responses were automatically collected anonymously and stored securely in SurveyMonkey’s online database. Partial responses were accepted and included in the results.

### Data analysis

Raw data was imported directly from SurveyMonkey into Excel (Microsoft, Version 16.16.24). Descriptive statistics were calculated for all survey responses. Mean, median and modes were calculated for all questions with a Likert scale to identify the most accurate and representative majority response. Based on survey responses to location of virtual care, subjects were classified as either academic/hospital base or private/community based. Responses between community and academic physicians were compared to determine if there were any differences in responses. Answers were analyzed using nonparametric and independent two tailed student’s t-test where appropriate. Statistical significance was defined as *p* < 0.05. Stata (Version 14.1) was used for all statistical analyses.

## Results

The survey link was sent to 61 practicing Canadian rhinologists. Over the survey collection period, there were a totally of 43 responses with a response rate of 70.5%. The survey had a completion rate of 88%. The incomplete questions represented the free text “additional comment” questions (Q31, Q32). The average time to complete the survey was less than seven minutes. Results are available in detail in the supplemental materials.

A variety of platforms were used by the respondents. 100% of respondents used the telephone for virtual visits and this was the primary platform for 95.2% of respondents. Other platforms used for virtual visits included hospital approved Telehealth (21.4%), Facetime (4.8%), Zoom (4.8%) and Medeo (4.8%). Physicians performed virtual care from a variety of practice settings and most physicians used more than one location. The majority of respondents performed their virtual visits from home (57.1%). Based on the location of their primary practice, 46.5% of the physicians were community based and 53.5% were academic/hospital based (Table 1).

**Table 1:** Platform for Virtual Care

Q1. Which platforms do you use to delivery virtual care during the SARS-CoV-2 pandemic? (select all that apply)	
Telephone	100%
Facetime	4.8%
Telehealth	21.4%
Zoom	4.8%
Other: Medeo	4.8%
Skype, Google Hangouts	0%
**Q2: If you use more than one platform, which do you use most commonly?**	
Telephone	95.2%
Telehealth	2.4%
Zoom	2.4%
**Q3: Do you perform virtual care from your: (select all that apply)**	
Home	57.1%
Private Office	45.2%
Hospital Based Office	52.4%

### History

The majority of respondents reported that history taking was equivalent during a virtual visit to an in-person visit (74.4% strongly agree or agree). Over 90% of respondents reported that they could determine the history of presenting illness, past medical history and allergies as well in virtual care as traditional in-person care. Past surgical history, family history and social history were reported to be equivalent by 87 to 89% of participants. Medication history was the least reported area, with 79.5% of respondents reporting their history would be equivalent. The comments highlighted that in person, patients can provide a written list of medications that can be scanned or transcribed into the medical record. The free text question (Q31, Q32) reported that history taking was similar during a virtual visit, and doing so virtually may help limit in-person exposure time to patients.

### Physical examination

The physical examination was the area of a traditional appointment that was reported to be the most affected by virtual care. 88.3% of respondents strongly disagreed or disagreed that the exam was equivalent during virtual care. 66.7% reported that no aspect of the physical exam could be performed during virtual care to an equivalent level, while approximately 20% reported that the external nasal exam, the Cottle/modified Cottle maneuver and Teapot maneuver (for CSF rhinorrhea) could be performed adequately during virtual care. Approximately 10% reported that a general assessment, caudal septal assessment and cranial nerve exam could be performed equivalently during virtual care. The comments in this section did emphasize that the exam is limited to inspection only. 83.7% agreed or strongly agreed that their physical exam was limited because of the lack of nasal endoscopy. In the free text question (Q31, Q32), 89.7% of the responses reported the lack of nasal endoscopy as a significant limitation of virtual care.

### Diagnosis, treatment, testing

Many of the respondents reported that their ability to diagnose disease, order investigations, and initiate medical and non-medical therapy was similar to in person care during virtual visits. When assessing whether virtual care was equivalent to in-person care for identifying a patient’s diagnosis, the majority of respondents reported virtual care was less effective (58.1% disagree or strongly disagree). Approximately half reported reported ordering investigations was similar during by virtual care. 58.1% of the participants reported their ability to initiate medical therapy was equivalent, and 48.8% reported that their ability to prescribe medication was equivalent. In the free text question (Q31, Q32), missing a diagnosis and the inability to perform a biopsy were both reported as limitations of virtual care.

### Results of investigations

The vast majority of respondents felt virtual care was equivalent to in-person care for reviewing imaging, lab work and tissue pathology. 81.4% agreed or strongly agreed that virtual care was equivalent for reviewing imaging, and 86.1% agreed or strongly agreed that virtual care was equivalent for reviewing lab work and pathology results. Of the free text comments that were submitted (Q31, Q32), respondents reported feeling that virtual care was well suited to reviewing results with patients.

### Communication

34.9% of respondents reported their ability to establish rapport was equivalent to in-person appointments. There was a fairly even split in opinions on whether explaining diagnosis and treatment was equivalent during virtual care, with approximately 30% agreeing, 30% neutral and 30% disagreeing. The majority of participants reported that their ability to consent patients for surgery during a virtual visit was not equivalent and 60.4% would not operate on a patient without meeting them in person first.

### Satisfaction and quality of care

When examining overall satisfaction and perception of quality of care, most respondents felt virtual care was not equivalent to in-person care. 25.6% reported that they believed patient satisfaction during virtual care was equivalent to in-person care. Few respondents (7.0%) felt the quality of care during virtual care was equivalent to in-person care and only 11.6% felt physician satisfaction was equivalent.

### Types of patient encounters

Several questions assessed what type of patient encounters may be best suited to virtual care. The virtual appointment type that was felt to be most equivalent to in-person appointments was non-urgent follow up visits (54.8% agree or strongly agree). Non-urgent new patient appointments were reported to be equivalent by 33.4% (agree or strongly agree) of respondents. Urgent patients (new and follow up) were only thought to be equivalent by 16.7 and 14.3% of respondents, respectively. Virtual visits were reported to be equivalent to in-person care in adult by 26.2% of respondents. Pediatric virtual visits were thought to be equivalent by 9.5%. Overall, non-urgent new and follow up visits were identified as the most appropriate for virtual care.

### Chief complaints

**Fig. 1:**
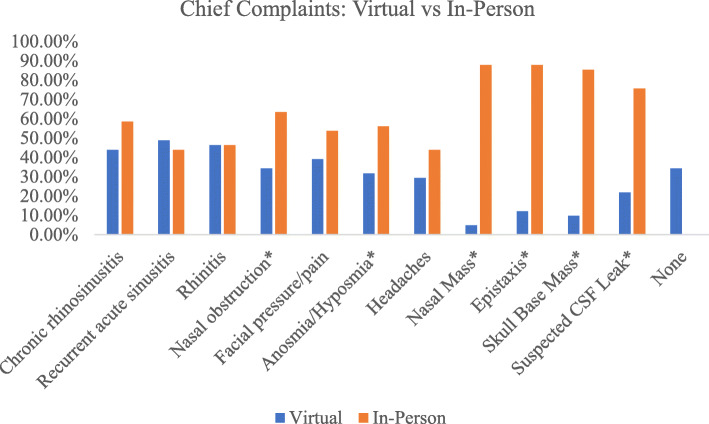
Chief Complaints: Virtual versus In-person visits

Participants were asked to select chief complaints they felt they could assess, diagnose, and initiate treatment for during a virtual visit, as well as those they felt would be better assessed in-person. Responses were compared to determine which chief complaints the respondents felt could be assessed appropriately in virtual visits, versus those they thought were better suited to in-person visits. Nasal obstruction, anosmia/hyposmia, nasal mass, epistaxis, skull base mass and suspected CSF leak were reported to be better assessed during in-person assessments by the majority of respondents (*p* < 0.05) (Fig. 1). 34.2% of participants thought no chief complaints could be assessed in a virtual visit to an equivalent level however, there was no statistical difference in the responses for virtual versus in-person assessment for chronic rhinosinusitis, recurrent acute rhinosinusitis, rhinitis, facial pressure/pain and headaches (*p* > 0.05), suggesting these presentations may be well suited to virtual visits.

## Discussion

This survey based cross-sectional study of Canadian rhinologists assessed the primary modalities of virtual care, the perceptions on how the areas of a traditional appointment compare to virtual visits, and what types of presentations are well suited to virtual care. The respondents highlighted the ability to obtain a history virtually as a strength of virtual care. They also identified virtual care to be equivalent to in person care when explaining diagnosis and treatment, and performing non-urgent new and follow-up assessments. The majority reported that virtual care was limited by the inability to examine and scope patients. The difficulty in establishing an effective doctor-patient relationship with virtual care alone was identified as another potential limitation. As we move into a hybrid of in-person and virtual care in response to the SARS-CoV-2 pandemic, it is important to identify the strengths and weaknesses of virtual care.

Prior to the 2020 SARS-CoV-2 pandemic, virtual care (telemedicine, telehealth) represented a very small area of medicine. Within a few weeks of the “stay at home” orders and strictly enforced physical distancing requirements in North America, virtual visits went from constituting 5% all patient visits, to over 90% [2]. There has been an associated increase in the publications related to virtual care, though prior to this the research into telemedicine was limited. A 2015 Cochrane review identified 93 trials comparing telemedicine to traditional care, and noted it was primarily used to survey chronic disease for patients located at a distance [3]. Much of the emerging literature in otolaryngology on virtual care emphasizes implementation (billing, documentation, medicolegal issues, practice patterns) [4–7]. Some of the literature has focused on both patient and physician perceptions of virtual care [5, 8, 9]. There is no literature assessing how virtual care performs compared to traditional in-person appointments in otolaryngology.

A variety of platforms are used to provide virtual care (Table 1), but the vast majority (95.2%) used simple phone calls as their primary virtual platform. While the telephone is limited by its lack of face-to-face interaction, other research on video calls during the pandemic have highlighted issues with video connectivity and lag affecting communication [10]. There are also some issues with accessibility – a recent study showed that adults > 65 years of age were less likely to have a telehealth visit [11]. While the telephone is not a previously accepted form of virtual care, this has shifted during the pandemic. The ease of use and accessibility of the telephone could explain why this is the majority platform for virtual care at this time – it requires no additional software installation, no IT support and patient phone numbers are already included in the majority of patient charts.

When asked about the components of a traditional in-person appointment compared to a virtual appointment, history taking and reviewing results (lab work, imaging, pathology) were reported to be equivalent to in-person appointments by the majority of respondents. These are the sections of a traditional appointment which are more reliant on verbal communication. These are also typically the most time-consuming components of a patient visit. The duration of time spent with a COVID positive individual is related to the likelihood of disease transmission, and virtual care helps limit face to face exposure time. This may help limit physician exposure in the case of an unknown interaction with an infectious COVID positive patient. Physical examination, diagnosis, treatment and ordering investigations were reported to be limited by virtual care. The inability to perform nasal endoscopy was reported to be a significant limitation by the majority of participants (83.7%).

Patient communication, overall satisfaction and quality of care were reported to be limited by virtual care in this study. This is in keeping with the result of other recent literature that has highlighted that patients are less satisfied with virtual care [10]. A recent study of American Rhinologic Society members reported that 74.4% participants had some psychologic or emotional impact from the current pandemic, though did not attribute this to any specific factors. However, this form of care is novel to the majority of patients and physicians, and there is documentation that people experience more fatigue during virtual calls and conferences (“Zoom Fatigue”), due to the loss of facial expressions, body language and the audio-video lag associated with most platforms [12]. This may help explain why the respondents may be less satisfied with this modality. However, there is some evidence that suggests many patients are satisfied with virtual care. While novel, it allows them access to their physicians that they otherwise would not have during this time [8, 9].

Currently there are no clear guidelines about which patients are best suited to in-person or virtual visits. Previously, telemedicine was largely limited to follow up visits for patients with chronic illnesses [3]. Participants in this study identified that non-urgent follow up visits were felt to be equivalent to in-person care and non-urgent new consultations were thought to be equivalent by 33.4% of participants. The majority of participants reported virtual visits were not equivalent for urgent patient concerns. Chief complaints such as suspected chronic rhinosinusitis, recurrent acute rhinosinusitis, rhinitis, facial pressure/pain and headaches were reported to be equivalently assessed by virtual care. Nasal obstruction, nasal mass, epistaxis, skull base mass and suspected CSF leak were reported to be better assessed by in-person assessments. The accumulated opinions and expertise of the respondents may help guide clinicians when determining which patients to prioritize for in-person appointments.

Overall, the goal of this study was to better understand the opinions of Rhinologists regarding how virtual care functions and can be integrated into a rhinologic practice. There are some limitations which must be acknowledged. This is a survey study, which are inherently subject to recall bias and reflect the opinions of respondents. There is also a component of selection bias in the respondents. The study participants are Rhinologists – as such, the results of this study may not be generalizable to general Otolaryngologists or other subspecialists. This study is strengthened by the excellent response rate. This study is also novel in that it examines physicians’ perspectives on the strengths and weaknesses of virtual care, which can help provide insight for physicians’ trying to navigate this new modality of care.

## Conclusion

The use of virtual care has increased substantially in response to the necessity of physical distancing during the SARS-CoV-2 pandemic. As we move to a hybrid of in-person and virtual care model, it is important to understand the limitations of this new treatment modality, and where it can work most effectively. Overall, virtual care is reported to be well suited to history taking and reviewing results with patients. Non-urgent new and follow up presentations are likely the most appropriate for virtual care. Chief complaints that are non-urgent in nature and less dependent on physical examination are well suited to virtual care. The inability to perform a physical exam with nasal endoscopy is considered to be a limitation of virtual care in Rhinology. These results clarify the role of virtual care in Rhinology at this time, and will help physicians prioritize patients for either in-person or virtual care.

## Data Availability

All data generated or analyzed during this study are included in this published article and its supplementary information files.
